# In What Cases Shall the Medical Examiner Decline to View a Dead Body?

**Published:** 1882-05

**Authors:** Alfred Hosmer

**Affiliations:** Medical Examiner; Watertown


### IN WHAT CASES SHALL THE MEDICAL EXAMINER DECLINE TO VIEW A DEAD BODY? By Medical Examiner ALFRED HOSMER, M.D., of Watertown.*

To the rule laid down I have in practice made an exception when a notice has been sent by a magistrate or municipal officer of high grade. I have then entered upon a medical examination without preliminary questions, and it is a curious fact that in all such instances I have found myself dealing with a kind of inverted cone, so that the further down the investigation proceeded the nearer it has come to nothing.

"Upon the view of the dead bodies of such persons only as are supposed" etc. The word "only" is here used in a strong restrictive sense, and contains the prohibition which limits the official competence and jurisdiction of the medical examiner. Unless there be a supposition of violence his commission confers upon him no authority and he is powerless.

In deciding what is the significance of the word "persons," I shall take the liberty of referring you to an instructive paper,* written in answer to the question "What constitutes the dead body of a person?" and presented to this Society at the February meeting. The conclusions to which the writer came, after careful study and consultation, interests us now only so far as they relate to what of humanity is not included in the definition of "persons," as that term is understood and accepted by those who interpret law, and explain the intent of its equivocal portions. According to the indisputable authorities whom he quotes, judicial voices all agree in the declaration that a child is not born until it is entirely extruded from the maternal body, and that, although it may have breathed at some point in the partuient passage, if it be still at the instant of complete expulsion it has never become a person; and, therefore, cannot have been the subject of such violence as the medical examiner is qualified to recognize and certify to. No child known to be dead-born can be inspected in the sense of viewing the dead body of a person. But the question at once arises as to what course the medical

* B ston Medical and Surgical Journal, vol. ciii., p. 200.

examiner shall pursue in the event of the discovery, through the improper disposition of the body of a child born prematurely or at term, respecting which no information can be obtained. For a portion of them, Medical Examiner Holt lays down the following rule: "It would, perhaps, only be safe to conclude that all fœtuses born in the first half of pregnancy cannot live for any period, and the body of such an one cannot be considered the dead body of a person within the meaning of the law."

According to Caspar the maximum length of a fœtus at the end of the twentieth week of utero-gestation is twenty-eight centimeters. The application of a metric measure will determine with sufficient accuracy whether in a particular instance the medical examiner is dealing with a person or a thing. In other words he must decline to view a fœtal body unless it be more than twenty-eight centimeters long.

It appears from the Executive Board for 1879 * that in thirty-eight per cent. of all the views held the cause of death was found to be a natural one. Where three succumbed to violence, two yielded to nature. Can it be that in so large a proportion of the cases there was so much obscurity and mystery as to justly excite a suspicion of unlawful agency? Is it not possible that the office of medical examiner, like the drug last added to the materia medica, has been perverted through its application to conditions for which it was never intended? If this mistake has been committed, there was during the first few months that followed his institution an apology for it in the deficient condition of our statutes. An act † of the legislature of 1878 is the first that has

---

* Boston Medical and Surgical Journal, vol. ciii., p. 265.

† ACTS OF 1878, CHAPTER 174. SECT. 1. No human body shall be buried, or removed from any city or town, unless a proper certificate has been given by the clerk or local registrar of statistics to the undertaker or sexton, or person performing the burial, or removing the body. This certificate shall state that the facts required by chapter twenty-one of the General Statutes have been returned and recorded; and no clerk or local registrar shall give such certificate or burial permit until the certificate of the cause of death has been obtained from the physician, if any, in attendance at the last sickness of the deceased, and placed in the hands of said clerk or local registrar; *provided*, that in those cities and towns where local boards of health have been established, the certificate of the cause of death shall be approved by such board before a permit to bury is given by the registrar or clerk. Upon application, the chairman of the local board of health or any physicsan employed by any city or town for such purpose, shall sign the certificate of the cause of death to the best of his knowledge and belief, if there has been no physician in attendance. He shall also sign such certificate, upon application, in case of death by dangerous contagious disease, or in any other event when the certificate of the attending physician cannot for good and sufficient reasons be easily enough obtained. In case of death by violence, the medical examiner attending shall furnish the requisite medical certificate. Any persou violating the provisions of this section shall be punished by a fine not exceeding twenty-five dollars.

ever authorized any one to certify to the cause of death whenever there has been no medical attendant during the last illness. Having made a general provision, it excepts cases of violence, and refers them to the medical examiner for certification. It thereby intimates that the simple fact that a man has died without medical supervision is not a sufficient reason for demanding a view; that it does not require or even permit the medical examiner to hold one.

"But nothing herein shall prevent any medical examiner from acting as such in any part of his county." This provision forms the concluding portion of the sixth section of the law to which we owe our official being. It is simply a negation, and conveys the idea that the medical examiner has full legal liberty to exercise his office throughout his county irrespective of the district lines originally established by commissioners. Without violating any statute a physician may render professional service to every patient who seeks it, yet there is a rule of etiquette, dictated by the sentiments of honor, justice and propriety, not to speak of the less worthy consideration of self-protection, which restrains the average medical man, and admonishes him not to interfere in work already assigned to another. This principle should guide the medical examiner in his conduct towards official neighbors. Human nature is tenacious of its lawful possessions, it is jealous and intolerant of seeming intrusion. Provoke collision and coöperation is at an end. The success of this new system will not attain the highest possible degree unless its essential instruments act in cordial harmony. We can give no better proof of our devotion to the cause which we have espoused, and to which the acceptance of our commissions has committed us, than by cultivating and maintaining among ourselves relations, not simply of mutual good-will and confidence, but of active friendship. We should adopt a policy which is rendered safe and simple by the exclusion of all doubts and questions as to propriety. Therefore I hold the opinion that a medical examiner should decline to hold a view outside of the district for which he has been appointed unless the medical examiner whose district he enters either requests to be relieved from duty in a particular instance, or is so situated that his services cannot be obtained within a period of time which is reasonable when considered with reference to the necessities of a given case.

Practice is the acknowledged route to perfection, and the novitiate is terminated only by the exercise and action which constitute experience, and confer new ability. For his training and instruction, for the gratification of that sense which appreciates and enjoys the curious and the interesting, the medical examiner needs all the opportunity and all the material which his district can possibly furnish. He is defrauded and retarded in his official growth whenever that to which he alone has a claim is converted to the use and advantage of another.

The medical examiner required to be "learned in the science of medicine," must by a physician, and is under a double obligation to be of good repute. Even if his ideas of duty are less severe than those which have found expression here, his conduct should be regulated by those principles, a strict adherence to which has made the name of medicine so suggestive of intelligence, moral worth and public spirit.

